# High-grade, metastatic disease, and adjuvant radiotherapy are independent prognostic factors for progression-free survival in patients with solitary fibrous tumors

**DOI:** 10.1093/noajnl/vdaf077

**Published:** 2025-04-17

**Authors:** Jan Paul Alker, Ramin Rahmanzade, Thomas Held, Christel Herold-Mende, Andreas Unterberg, Felix Sahm, Sandro Manuel Krieg, Gerhard Jungwirth

**Affiliations:** Department of Neurosurgery, Heidelberg University, Heidelberg, Germany; Department of Neuropathology, Heidelberg University, Heidelberg, Germany; Department of Radiation Oncology and Radiotherapy, Heidelberg University, Heidelberg, Germany; Department of Neurosurgery, Heidelberg University, Heidelberg, Germany; Department of Neurosurgery, Heidelberg University, Heidelberg, Germany; Department of Neuropathology, Heidelberg University, Heidelberg, Germany; Department of Neurosurgery, Heidelberg University, Heidelberg, Germany; Department of Neurosurgery, Heidelberg University, Heidelberg, Germany

**Keywords:** central nervous system tumors, hemangiopericytomas, metastatic disease, radiotherapy, solitary fibrous tumors

## Abstract

**Background:**

Solitary fibrous tumors (SFTs)/hemangiopericytoma are rare central nervous system tumors exhibiting high recurrence rates and the ability to metastasize. This study evaluated SFT prognosis and survival outcomes, focusing on the 2021 WHO classification.

**Methods:**

A retrospective study was conducted on 49 patients who underwent SFT resection in our Neurosurgery Department between 2001 and 2023. Data were analyzed regarding sex, age, WHO grade at time of diagnosis and reclassified according to the 2021 WHO classification, tumor localization, resection grade, tumor size, adjuvant therapy, progression-free (PFS), and overall survival (OS). Kaplan-Meier survival analyses were conducted to evaluate OS and PFS, and Cox regression analyses were performed to assess prognostic factors.

**Results:**

Cohort median age was 54 (22-86) years with a female predominance of 1.22. The median follow-up was 46 (0-307) months. Primary SFTs were mainly located in the supratentorial region, followed by the infratentorial region and spine. Initially, 10% of primary tumors were graded as WHO grade 1, 49% as grade 2, and 18% as grade 3. Reclassification to WHO 2021 downgraded 65% of tumors. The five-year PFS and OS were 41.5% and 100%, respectively. In total, 41% of patients had local recurrent disease and 20% were metastatic. In univariate analyses, WHO grades, younger age (< 54 years), sex, and adjuvant radiotherapy were associated with survival. In multivariate analyses, WHO grade 3, metastatic disease, and adjuvant radiotherapy were independent PFS prognostic factors.

**Conclusion:**

Our data shows that WHO grade 3, metastatic disease, and adjuvant radiotherapy are independent PFS factors in SFTs.

**Key points**

- Reclassification of SFTs to WHO 2021 led to the downgrading of most SFTs without altering survival data.
- WHO grade 3, metastatic disease, and adjuvant radiotherapy are independent prognostic factors for PFS in patients with SFTs.

**Importance of the Study**

This study comprehensively evaluates prognostic factors and survival outcomes in patients with solitary fibrous tumors (SFTs), rare central nervous system tumors known for their high recurrence and metastatic potential. By retrospectively analyzing data from 49 patients treated between 2001 and 2023, this study highlights the impact of the 2021 WHO reclassification on tumor grading and subsequent survival predictions. Key findings indicate that high WHO grade, metastatic disease, and adjuvant radiotherapy are significant independent prognostic factors influencing progression-free survival. This study provides valuable insights into the clinical management of SFTs, emphasizing the need for careful consideration of tumor grading, cancer staging, and therapeutic strategies to improve patient outcomes.

Solitary fibrous tumors (SFTs) are rare tumors of the central nervous system.^1^ They exhibit radiological characteristics similar to meningiomas, including dural attachment and homogeneous contrast enhancement.^2,3^ However, whereas the incidence of meningiomas is around 8.3 per 100 000 individuals, SFTs are significantly less common, occurring at a rate of just 0.06 per 100 000 individuals, making them more than 100 times rarer.^4^ Clinically, SFTs are known for their aggressive behavior, high recurrence rates, and potential to metastasize to other areas of the central nervous system or distant organs, particularly the liver, lung, and bone.^5,6^ The 2016 World Health Organization (WHO) classification of central nervous system tumors recognized hemangiopericytomas and SFTs as a single entity.^7^ In the revised 2021 WHO guidelines, the term “hemangiopericytoma” was eliminated and SFTs were classified into 3 grades based on mitotic activity and necrosis.^8^ SFTs are mesenchymal, non-meningothelial tumors characterized by the presence of a *NAB2* (NGFI-A binding protein 2) and *STAT6* (signal transducer and activator of transcription 6) gene fusion.^9^

Due to the rarity of these tumors, most published studies include only a small number of patients.^5,6,10–13^ The largest series comprised 40 or more patients^14,15^; however, most were based on the same patient cohort of the Surveillance, Epidemiology, and End Results Program (SEER).^5,6,10–13^ This limitation, in conjunction with recent revisions to the WHO classification, introduces uncertainties regarding the optimal therapeutic strategies for patients with SFTs, particularly regarding adjuvant therapies following surgical resection.

This study provides insights into the prognostic factors of a large SFT patient cohort by analyzing demographics, tumor localization, resection grade, tumor volume, adjuvant therapy, WHO grade at the time of diagnosis and reclassified according to the 2021 WHO classification, progression-free (PFS), and overall survival (OS). Hereby, we demonstrated that WHO grade based on the 2021 WHO classification, metastatic disease, and adjuvant radiotherapy are independent prognostic factors for PFS in SFT patients.

## Methods

### Clinical Data

Our institutional database was screened for patients who underwent surgical resection of an SFT at the Department of Neurosurgery of University Hospital Heidelberg, Germany, between 2001 and 2023. Institutional review boards approved this study following the Declaration of Helsinki; written informed consent was obtained from all patients. Demographic and tumor-related factors, including tumor location, size, WHO grade, and treatment-related and outcome data, were collected retrospectively from medical chart review and magnetic resonance imaging (MRI) studies. The WHO grade at initial diagnosis was determined based solely on pathologist reports available at the time of surgery. Tumors were classified according to the WHO classification in effect at the time of diagnosis. Specifically, 2.6% of the tumors were graded using the second WHO classification, 7.8% with the third, 84.4% with the fourth, and 5.2% with the fifth WHO classification. Tumor volumetry was performed using iplannet software from Brainlab. Available tumor tissues (74/114 of all tumors, including local recurrences and central nervous system metastasis) were reclassified according to the 2021 World Health Organization Classification of Tumors of the Central Nervous System by a board-certified neuropathologist (Department of Neuropathology, University Hospital Heidelberg, Germany). The extent of resection (EOR) was determined based on surgical reports and/or early postoperative MRI scans. In 79.5% of cases, both sources were available, while only one option was available in 20.5% of cases. In instances of conflicting data between MRI scans and surgical reports, MRI scans were prioritized to determine the EOR. EOR was categorized as gross total resection (GTR, Simpson °I-III) or subtotal resection (STR, Simpson °IV-V).^16^ PFS was defined as the interval between the first surgery and the radiological or clinical detection of local tumor progression or metastasis. Follow-up was defined as the interval from the first surgery until the final patient contact. T1-weighted post-contrast imaging was used for precise tumor delineation. The gross tumor volume (GTV) incorporated potential microscopic spread, considering all available information, including surgical reports and pre- and postoperative imaging. Depending on the irradiation modality, an isotropic margin of 1-5 mm was added to the planning target volume (PTV) to account for geometric uncertainties and physical inaccuracies of the beam, in line with physics recommendations.

### Statistical Analyses

Statistical analyses were performed using the Kruskal–Wallis test, Log-rank test, and Cox regression with GraphPad Prism software (Vers. 10.2). *P*-values < .05 were considered significant (*, *P* < .05; **, *P* < .01; ***, *P* < .001).

## Results

### Clinicopathological Characteristics of SFT Patients

**Figure 1.**
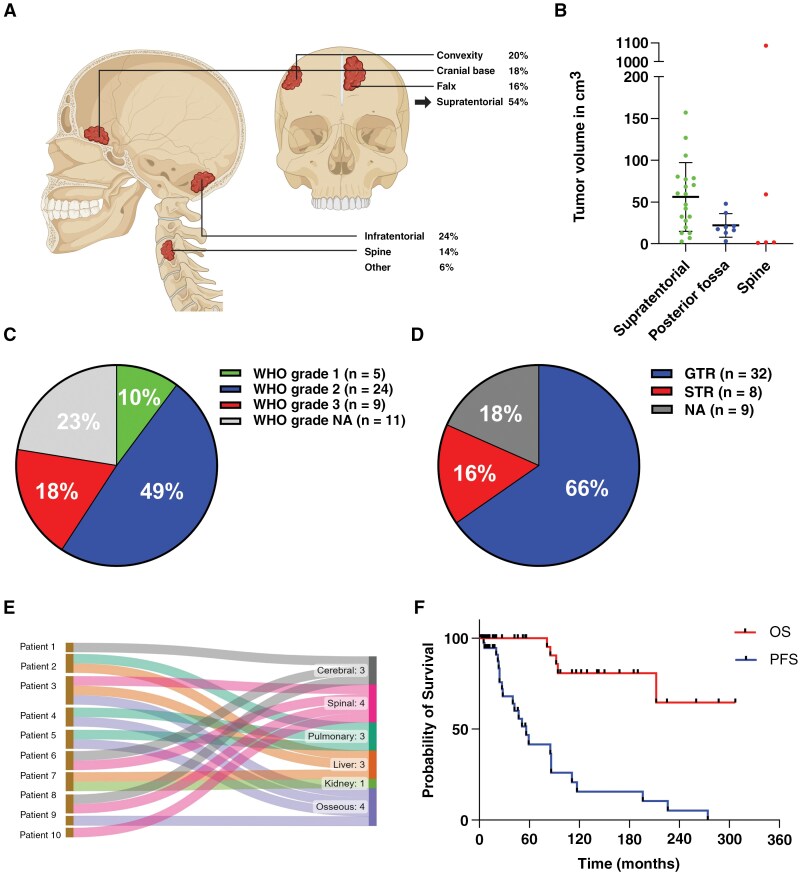
Clinicopathological characteristics of the patient cohort. **(A)** The localization and distribution of central nervous system solitary fibrous tumors (CNS-SFTs) at initial diagnosis. **(B)** Tumor volumes are categorized by location and measured in preoperative contrast-enhancing T1 MRI scans. **(C)** Pie chart illustrating the distribution of WHO grades for primary tumors at the time of diagnosis. (**D)** Pie chart showing the proportions of gross total resection (GTR) and subtotal resection (STR) during primary tumor surgery. (**E)** Sankey plot displaying all patients with metastasis. (**F)** The Kaplan-Meier plot displays overall and progression-free survival for primary tumors. PFS: Progression-free survival, OS: overall survival, NA: not available.

The patient cohort analyzed in this study included 49 patients, with a female predominance of 1.22. The clinicopathological characteristics are summarized in Table 1. The median age at initial diagnosis was 54 years (range: 22–86 years). Primary tumors were mainly located in the supratentorial region (54%), followed by 24% in the infratentorial, and 14% were spinal tumors. Supratentorial tumors were found mainly at the convexity (20%), followed by the cranial base (18%) and the falx (16%). Two SFTs (4%) were in the orbit (Figure 1A). The median tumor volume of primary tumors was 32.5 cm^3^ (range: 1.02–1085.7 cm^3^). Supratentorial tumors were larger than infratentorial and spinal SFTs (median tumor size 53.3 cm^3^, 18.8 cm^3^, and 8 cm^3^, respectively, Figure 1B). The largest spinal tumor infiltrated the chest wall and was resected together with our colleagues from the thoracic surgery department. Its presence significantly impacted the overall tumor size distributions (*P* = .09, Krustal–Wallis test). When this tumor was removed from the analysis, we observed a significant difference in tumor sizes across different localizations (Figure 1B, *P* = .0132, Krustal–Wallis test). At initial diagnosis, 10% (*n* = 5/49) of primary tumors were graded as WHO grade 1, 49% (*n* = 24/49) as grade 2, and 18% (*n* = 9/49) as grade 3 tumors (Figure 1C). The WHO grade was unavailable in 22% of tumors (*n* = 11/49).

**Table 1:** Clinical data of patients with solitary fibrous tumors (*n* = 49)

Clinical factors	Group	Patients	
		N	(%)
Sex	Male	22	45
	Female	27	55
Age at initial diagnosis (years)	Median	54	
	Range	22-86	
WHO grade at initial diagnosis (primary tumors)	WHO grade 1	5	10
	WHO grade 2	24	49
	WHO grade 3	9	18
	NA	11	23
WHO grade at initial diagnosis (recurrences)	WHO grade 1	2	5
	WHO grade 2	8	22
	WHO grade 3	20	54
	NA	7	19
WHO grade at initial diagnosis (metastasis—systemic and central nervous system)	WHO grade 1	0	0
	WHO grade 2	1	3
	WHO grade 3	11	35
	NA	19	61
WHO grade reclassified based on the 2021 WHO classification	WHO grade 1	26	53
	WHO grade 2	8	16
	WHO grade 3	1	2
	NA	14	29
Tumor volume in cm^3^ at initial diagnosis	Median	32.5	
	Range	1.02-1085.7	
Tumor localization at initial diagnosis	Convexity	10	20
	Falx	8	16
	Cranial base	9	18
	Infratentorial	12	24
	Spine	7	14
	Other/NA	3	6
Adjuvant radiotherapy after initial surgery	No	35	71
	Yes	13	27
	NA	1	2
Follow-up (months)	Median	46	
	Range	0-307	
Patients with extracranial metastasis	All	6	12
	Liver	3	6
	Lung	3	6
	Bones	4	8
	Kidney	1	2
Progression-free survival after initial	Five-year PFS		41.5
diagnosis (%)	Ten-year PFS		15.6
Overall survival after initial	Five-year OS		100
diagnosis (%)	Ten-year OS		80.7
Number of patients with a local tumor recurrence		20	41
Number of patients with intra- or extracranial metastasis		10	20
Number of patients with local tumor recurrence or metastasis		24	49
Number of patients who died during follow-up		5	10

NA: not available, OS: overall survival, PFS: progression-free survival.

GTR of primary tumors was achieved in 66% (*n* = 32/49) of patients, whereas 16% (*n* = 8/49) underwent STR (Figure 1D). In 9 cases (18%), the surgery reports or early MRI images were unavailable.

Two patients received preoperative embolization. A total of 27% (*n* = 13/49) of patients received adjuvant radiotherapy following initial surgery. Supplementary Table T1 provides detailed information about the radiated tumors including recurrences and extra CNS metastasis. Most patients received intensity-modulated radiation therapy (IMRT) with a total dose between 50 and -60 Gy delivered commonly in 30 fractions. Sixteen percent (*n* = 8/49) received experimental systemic therapies at later stages of tumor progression. During the follow-up, 41% (*n* = 20/49) of patients presented with one or more local recurrences, and 20% (*n* = 10/49) with intra- or extracranial metastasis (Figure 1E). Most local tumor recurrences were graded as WHO grade 3 (69%, *n* = 20), followed by grade 2 (27.6%, *n* = 8), and grade 1 (3.4%, *n* = 1) at the time of diagnosis. Of all local tumor recurrences with available data, the majority retained their WHO grade (75%, *n* = 18), whereas 25% (*n* = 6) presented with an increased WHO grade compared with the primary tumor using the original WHO classification. Extracranial metastases were detected in 12% (*n* = 6) of patients, spreading to the lung, liver, kidney, and bone (Figure 1E). The 5- and 10-year PFS rates of primary tumors were 41.5% and 15.6%, respectively. The 5- and 10-year OS rates were 100% and 80.7%, respectively (Figure 1F), with a median follow-up of 46 months (range 0–307 months).

### Reclassification to WHO 2021 Resulted in the Downgrading of Most SFTs

**Figure 2.**
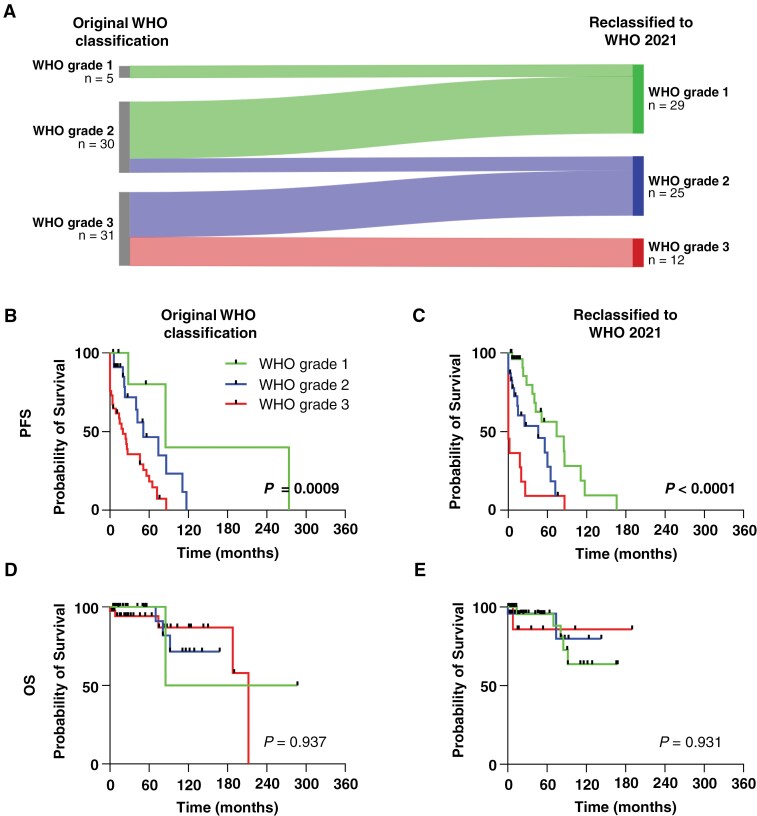
**The reclassification of solitary fibrous tumors (SFTs) according to 2021 WHO guidelines led to the downgrading of most tumors without affecting survival data. (A)** The Sankey plot illustrates the distribution of WHO classifications at the time of initial diagnosis (left side) and the subsequent reclassification based on the 2021 WHO guidelines (right side) for all tumors with both classifications available. **(B)** The Kaplan-Meier plot shows the progression-free survival of all SFTs based on the WHO grade at initial diagnosis or **(C)** reclassified to WHO 2021. **(D)** The Kaplan-Meier plot depicts overall survival based on the WHO grade at initial diagnosis and **(E)** according to the WHO grade following reclassification using the 2021 classification. Data includes primary tumors, recurrences, and central nervous system metastases. Prognostic significance was assessed using Log-rank (Mantel-Cox) tests. PFS: Progression-free survival. OS: overall survival.

All SFTs with available tissue (*n* = 73) were reclassified according to the 2021 WHO classification by our Department of Neuropathology. Notably, 65% (*n* = 43/66) of the tumors for which both classifications were available were subsequently downgraded (Figure 2A). Among the primary tumors, 71% (*n* = 22) were downgraded, while 29% (*n* = 9) maintained their original WHO grade. For tumor recurrences and metastases, 59% (*n* = 22) were downgraded, and 41% (*n* = 15) retained their initial grade. Significantly, no tumors were upgraded. Overall, the reclassification resulted in significant changes in tumor grades. Under the WHO 2021 classification, 29 SFTs were reclassified as grade 1, up from the previous count of 5. Similarly, 25 tumors were regraded as grade 2, down from the previous 30. Notably, the number of grade 3 SFTs decreased significantly from 31 to 12 after reclassification.

Next, we assessed the impact of the reclassification on PFS and OS for all tumors including metastases and recurrences. According to the WHO classification at the time of diagnosis, the median PFS of the cohort was 85 months for grade 1, 51 months for grade 2, and 27 months for grade 3 tumors (Figure 2B, *P* = 0.0009, Log-rank test). Following the implementation of the 2021 WHO classification, the median PFS decreased slightly to 74 months for grade 1 SFTs and 46 months for grade 2 SFTs (Figure 2C, *P* < 0.0001, Log-rank test). In contrast, the median PFS for grade 3 tumors dropped significantly from 27 months to 0 months after reclassification and was especially low for metastasis and recurrences (Supplementary Figure S1). However, survival rates for each WHO grade remained unchanged (Supplementary Figure S2). Furthermore, OS remained unchanged (Figure 2D, E).

In summary, the reclassification resulted in the downgrading of a significant number of SFTs without altering PFS or OS.

### Adjuvant Radiotherapy, Female Sex, and Younger Patient Age are Associated with Improved Survival in Univariate Analyses

**Figure 3.**
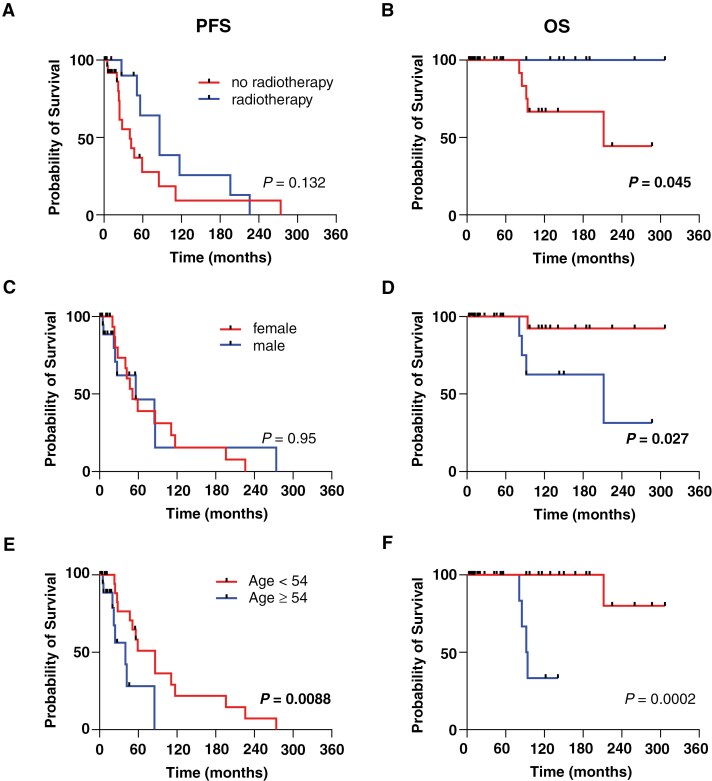
Univariate analysis shows associations of radiation, sex, and age with survival. Kaplan-Meier plots illustrating progression-free survival (PFS) **(A)** and overall survival (OS) **(B)** based on the administration of adjuvant radiotherapy following initial resection. **(C)** Kaplan-Meier plots depicting PFS and OS **(D)** in relation to the patient’s sex. **(E)** Kaplan-Meier plots showing PFS and OS **(F)** according to the patient’s age at initial diagnosis. The patients were categorized into groups according to their median age. The prognostic significance was evaluated using Log-rank (Mantel-Cox) tests. PFS, progression-free survival; OS, overall survival.

Univariate analyses were conducted to determine the impact of factors such as patient sex, age, tumor location, volume, EOR, and adjuvant radiotherapy on survival (Figure 3).

Twenty-seven percent (*n* = 13) of the patients received adjuvant radiotherapy following their initial surgery. Among these, 46% had grade 3 tumors (*n* = 6), while 31% were diagnosed with grade 2 SFTs (*n* = 4) based on the original WHO classification. WHO grades were not available for the remaining 3 patients (24%). Notably, most patients underwent GTR (*n* = 8, 61.5%), and only one (7.6%) underwent STR. This highlights that the WHO grade, rather than EOR, significantly influenced the decision-making regarding radiotherapy at our institution. Remarkably, radiation led to a significant improvement in OS, whereas PFS also increased but not significantly (Figure 3A, B, *P* = .045, *P* = .132, respectively, Log-rank test). The median OS for patients who received radiotherapy could not be determined, whereas it was 212 months for patients who did not undergo radiotherapy. The median PFS of patients who received radiotherapy was 86 months, in contrast to 40 months for those who did not.

Next, we evaluated the effect of sex on survival (Figure 3C, D); OS was significantly higher in female patients (*P* = .0027, Log-rank test), while PFS was unaltered.

Furthermore, patients who were younger than 54 years (median age of the cohort) had a significantly increased PFS (*P* = .0088, Log-rank test) and OS (*P* = .0002, Log-rank test) in comparison with older patients (PFS: 86 vs. 40 months, OS: unavailable vs. 93 months, respectively).

Tumor location, volume, and EOR did not impact the PFS or OS of patients with SFTs (Supplmentary Figure S3).

In summary, adjuvant radiotherapy, female sex, and younger patient age may be associated with improved OS, and younger patient age also with increased PFS.

### WHO Grades and Adjuvant Radiotherapy are Independent Prognostic Factors in Patients with SFTs

**Figure 4.**
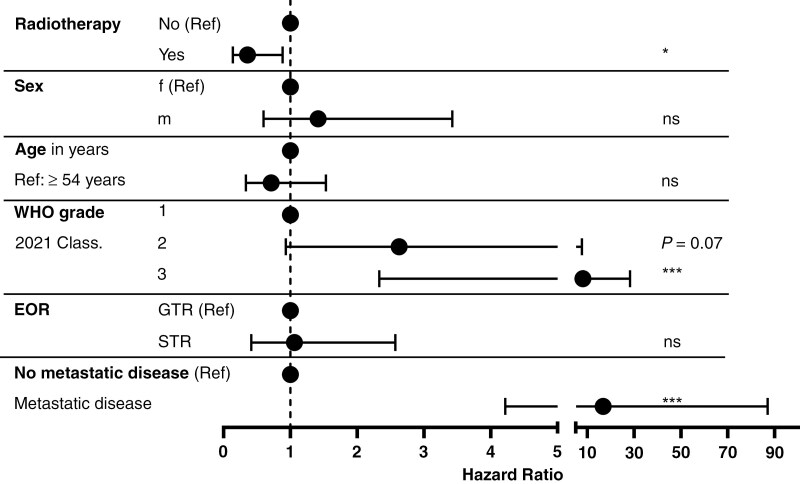
WHO grade 3, metastatic disease, and adjuvant radiotherapy are independent prognostic factors for PFS. The multivariate analysis assessed the effects of radiotherapy, sex, age, EOR, metastatic disease, and WHO grades on PFS using Cox regression. The results indicate that adjuvant radiotherapy hazard ratios (HR, 0.35, 95% CI 0.13 – 0.88, *P* = 0.02), metastatic disease (HR 16.75, 95% CI 4.21 – 87.02, *P* = 0.0002), and WHO grades influence PFS with an HR for grade 2 tumors of 2.62 (95% CI 0.93 – 7.69, *P* = 0.07) and of 8.14 for grade 3 tumors (95% CI 2.33 – 28.22, *P* = 0.0009). In contrast, male sex (HR 1.41, 95% CI 0.59 – 3.42, *P* = 0.42), age under 54 years (HR 0.71, 95% CI 0.33 – 1.53, *P* = 0.38), and subtotal total resection (STR; HR 1.06, 95% CI 0.41 - 2.57, *P* = 0.89) did not have a significant impact on progression-free survival.

To further explore factors influencing survival, we conducted multivariate analyses of all tumors including recurrences and metastases using Cox regression, incorporating variables such as patient age, sex, adjuvant radiotherapy, EOR, metastatic disease, and WHO grades according to the 2021 classification system (Figure 4).

A decrease in PFS was associated with WHO grade 2 (*P* = .07) and 3 tumors (*P* = .0009) compared with grade 1. Specifically, the hazard ratios were 2.62 (95% confidence interval [CI]: 0.93–7.69) for grade 2 and 8.14 (95% CI: 2.33–28.22) for grade 3 tumors (Figure 4). Additionally, adjuvant radiotherapy significantly improved PFS, with a hazard ratio of 0.35 (95% CI: 0.13–0.88, *P* = .028). Furthermore, metastatic disease was associated with a decrease in PFS with a hazard ratio of 16.75 (95% CI: 4.21–87.02, *P* = .0002).

In contrast, sex, EOR, and patient age did not correlate with changes in PFS. Furthermore, the multivariate analysis for OS did not reveal any significant impact from the variables considered.

Overall, WHO grade 3, metastatic disease, and adjuvant radiotherapy emerged as independent prognostic factors for PFS in patients with SFTs.

## Discussion

SFTs are rare central nervous system tumors known for their aggressive behavior and high recurrence rates. We retrospectively analyzed one of the largest cohorts—comprising 49 patients–and reclassified available tumor tissue according to the 2021 WHO classification. We found that most tumors were downgraded according to the new WHO 2021 classification. Furthermore, the study revealed that low WHO grades based on the 2021 WHO classification, younger age, female sex, and adjuvant radiotherapy were associated with better OS and younger patient age with increased PFS in univariate analyses. In multivariate analyses, WHO grade 3, metastatic disease, and adjuvant radiotherapy were independent prognostic factors for PFS. Overall, the findings underscore the importance of tumor grading, cancer staging, and postoperative radiotherapy in managing SFTs, advocating for further prospective studies to optimize patient care.

The demographics of our patient cohort, as well as tumor locations and rates of local recurrences and metastases, were consistent with findings from recent studies.^17–21^ According to a meta-analysis by Giordan et al., 43% of patients experienced local recurrences and 13%-17% developed metastases, varying according to tumor location.^17^ In our institutional cohort, we observed similar rates, with local recurrences occurring in 40% of patients and extra- and intracranial metastases in 20%. Our cohort’s 5-year and ten-year PFS rates were 41.5% and 15.6%, respectively, while the OS rates at 5- and 10-years were 100% and 80.7%, respectively. The 5- and 10-year OS rates reported for patients with SFTs were 79.4% and 76.6%, respectively.^17^

A substantial portion of tumors were downgraded following reclassification in accordance with the 2021 WHO classification. Under the 2016 WHO classification, SFTs were categorized based on their cellular architecture^7^: Grade 1 tumors contained a higher amount of collagen with fewer cells, grade 2 tumors had more cells and staghorn-like blood vessels, and grade 3 tumors exhibited 5 or more mitoses per 10 high-power fields. In contrast, the 2021 WHO classification defined grade 1 tumors as having less than 5 mitoses per 10 high-power fields, grade 2 tumors as having 5 or more mitoses per 10 high-power fields, and grade 3 tumors as having the same mitotic count as grade 2 tumors in conjunction with necrosis.^15,22^ As a result, it is not surprising that 65% of our tumors were downgraded, and no tumors were upgraded. Significantly, these changes did not influence PFS or OS outcomes in our cohort. Grade 1 SFTs presented the longest PFS, while grade 3 tumors had the shortest. Consequently, very few tumors remained classified as grade 3; those that remained grade 3 showed a markedly reduced PFS compared with those initially classified as grade 3 at diagnosis. This observation suggests that the new WHO classification may be more effective at identifying tumors associated with a poor prognosis. This highlights that a deeper molecular understanding of solitary fibrous tumors could enhance the accuracy of prognostic classifications. For instance, specific NAB2-STAT6 mutations have been shown to possess prognostic value regarding the potential for metastasis formation.^23^ Additionally, risk stratification models based on molecular markers, such as IDH1 mutations or PD-L1 expression, might predict tumor progression.^24^

Several studies explored the survival benefits of adjuvant therapy.^1,14,15,17–20,25–32^ In line with our findings, many reported no significant improvement in OS with adjuvant radiotherapy^18,19,25^ or that it was only observed in specific subgroups, such as patients with STR or GTR tumors.^14,15,20^ Conversely, some studies indicated that adjuvant radiation can enhance PFS and/or OS.^21,26–29,33^ Our findings suggest that adjuvant radiotherapy, particularly for high-grade tumors, improves PFS but does not significantly affect OS. In our institution, the decision to apply adjuvant radiotherapy is based on the WHO tumor grade. A meta-analysis of hemangiopericytoma patients—conducted before the 2016 WHO classification and aligning with high-grade SFTs classified under the 2016 or 2021 WHO guidelines—demonstrated improved OS following radiation, which supports our findings.^30^

In our study, the presence of metastatic tumors emerged as an independent risk factor for reduced progression-free survival. Due to the rarity of such cases, most studies on solitary fibrous tumors have not examined the impact of metastatic disease on survival. Among the studies that have analyzed the survival of patients with metastatic disease, results have been mixed—some report reduced OS in patients with metastasis,^17,18,34^ while others find no correlation between metastatic disease and OS.^35,36^ These inconsistent findings highlight the need for multicentric studies to expand patient cohorts and provide more definitive insights.

A limitation of this study is the median follow-up period of 46 months, which may affect the reliability of our 10-year survival rates, particularly regarding OS. This limitation could explain the slightly higher OS observed in our patient cohort compared to other studies.^17,19^

In conclusion, our analysis of one of the largest cohorts of SFTs highlights the significance of WHO grading, metastatic disease, and the role of adjuvant radiotherapy as independent prognostic factors influencing patient outcomes. The reclassification of SFTs per the 2021 WHO guidelines resulted in the downgrading of many tumors yet did not alter OS rates within our cohort. These findings underscore the urgent need to conduct randomized multicentric prospective studies to test the effectiveness of adjuvant radiotherapy and to enhance our understanding of this complex and challenging group of tumors.

## Supplementary Material

vdaf077_suppl_Supplementary_Material

## Data Availability

The data will be made available upon reasonable request.
