# Supplementary material for: High-grade, metastatic disease, and adjuvant radiotherapy are independent prognostic factors for progression-free survival in patients with solitary fibrous tumors
Source: Neurooncol Adv. 2025 Apr 17;7(1):vdaf077. doi: 10.1093/noajnl/vdaf077 (PMC12130975; doi:10.1093/noajnl/vdaf077)
Supplement: vdaf077_suppl_Supplementary_Material [file vdaf077_suppl_supplementary_material.docx]

**SUPPLEMENTARY FIGURES**

**
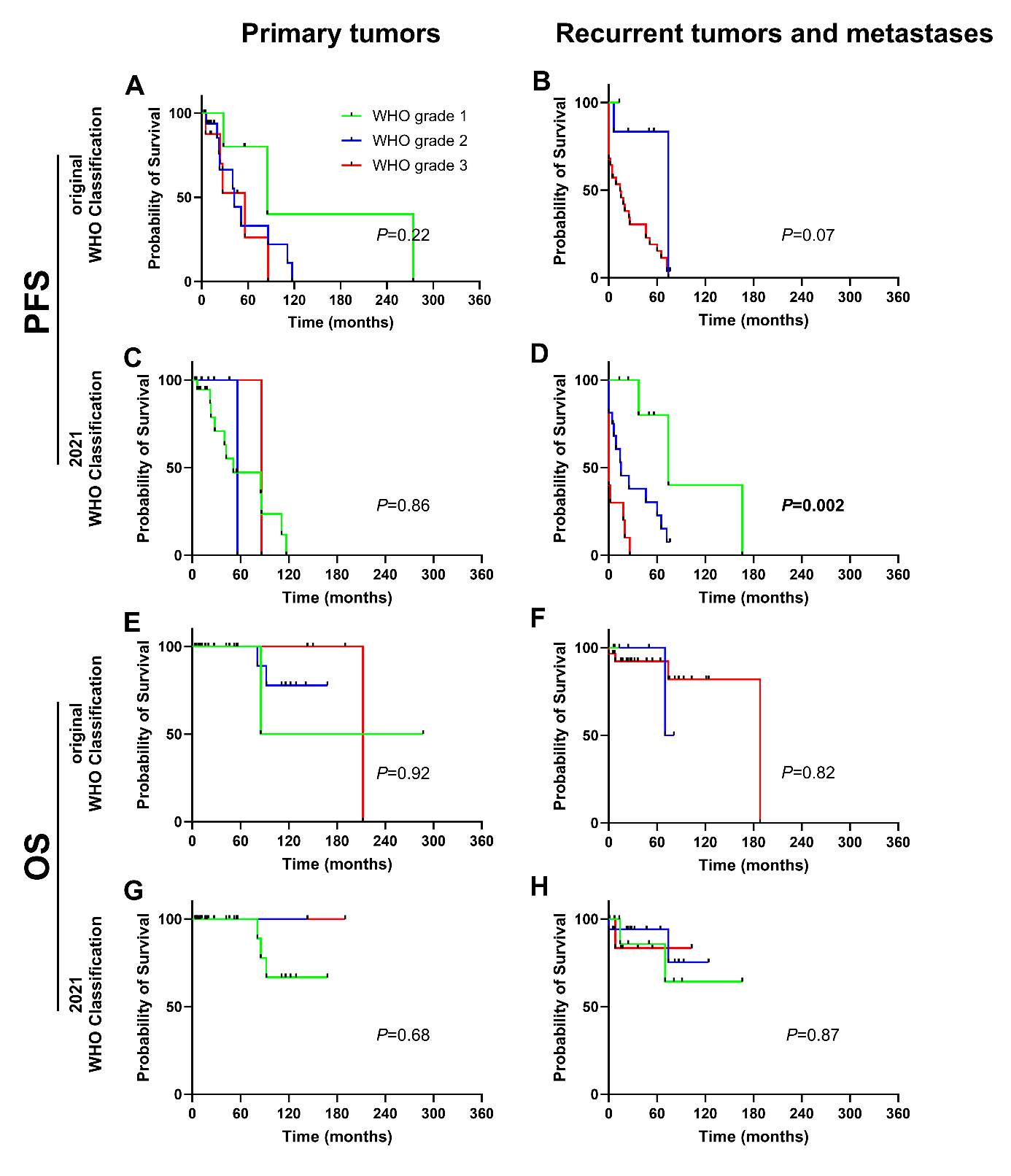
**

**Supplementary Figure S1 – Progression-free survival (PFS) and overall survival (OS) for primary tumors compared to recurrences and metastases based on the original and 2021 WHO Classifications**

Kaplan-Meier plots illustrate progression-free survival for primary tumors **(A&B)** and for metastases/recurrences **(C&D)**. Overall survival is presented for primary tumors **(E&F)** and for metastases/recurrences **(G&H)**, categorized according to WHO grades and WHO classifications. The prognostic significance was assessed using Log-rank (Mantel-Cox) tests. PFS: Progression-free survival, OS: overall survival.

**
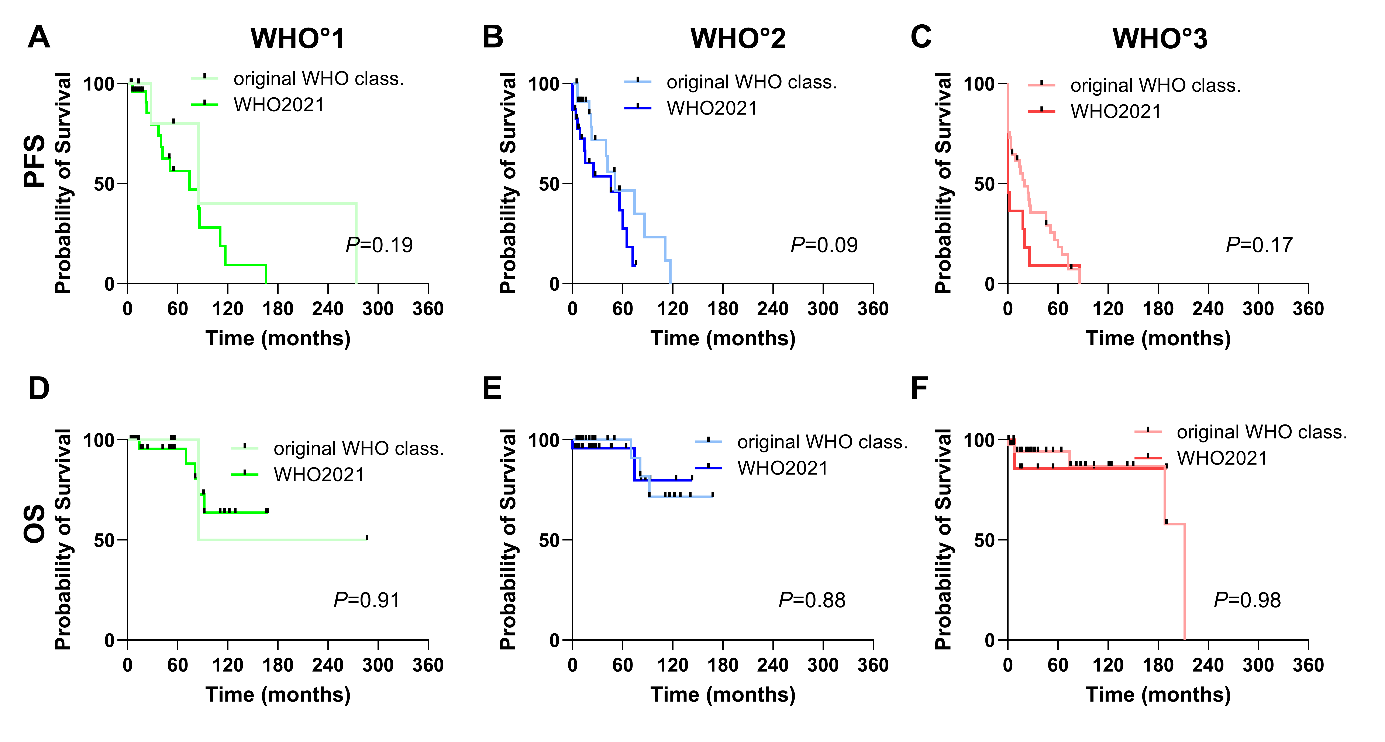
**

**Supplementary Figure S2 – Reclassification of tumors according to the 2021 WHO classification did not impact survival data.**

Kaplan–Meier plots illustrate progression-free survival **(A-C)** and overall survival **(D-F)** based on WHO grades and classification. Prognostic significance was assessed using Log-rank (Mantel-Cox) tests.

PFS: Progression-free survival, OS: overall survival.


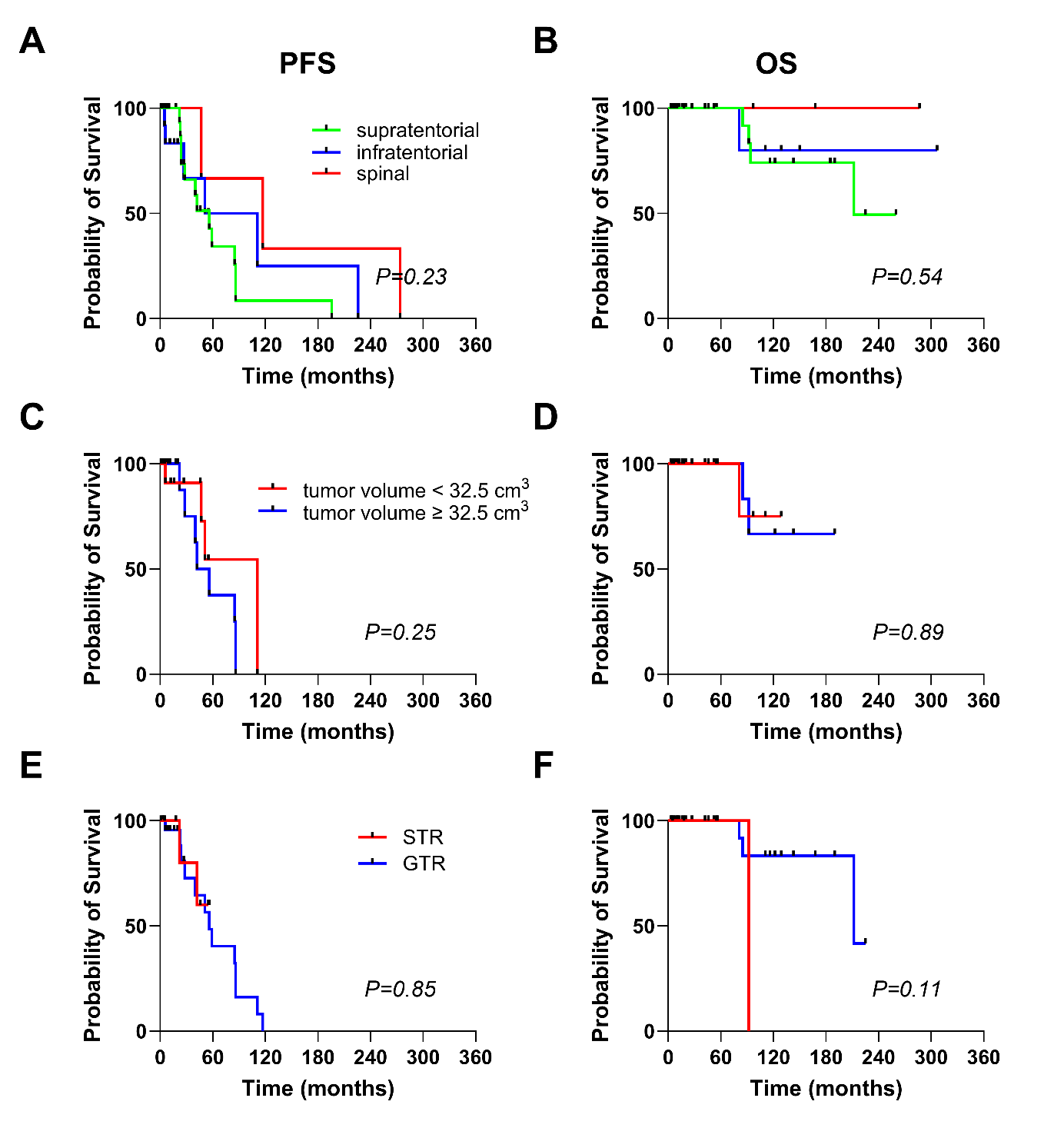


**Supplementary Figure S3 – Tumor localization, volume, and extent of resection did not impact survival.**

Kaplan–Meier plots show progression-free survival and overall survival associations with tumor localization **(A&B)**, tumor volume **(C&D)**, and extent of resection **(E&F)**. The patients were categorized into groups according to their median tumor volume, gross or subtotal resection (GTR, STR), or tumor localization (supratentorial, infratentorial, spinal). Prognostic significance was determined using Log-rank (Mantel-Cox) tests. PFS: Progression-free survival. OS: overall survival.

**SUPPLEMENTARY TABLES**

**Supplementary Table T1:** Clinical data of tumors receiving radiation (n=41)

| **Clinical factors** | **Group** | **Patients** | |  |
| --- | --- | --- | --- | --- |
|  |  | N | (%) | |
| Tumor type | Primary tumor  Recurrence / CNS metastasis  Extra-CNS metastasis | 13  20  8 | 31.7  48.8  19.5 | |
| Technique/modality | IMRT (Photons)  C12-Ions  Protons  He-Ions  Stereotactic (Photons)  IGRT (Photons)  VMAT (Photons)  Unknown modality | 10  7  2  1  3  1  1  16 | 24.4  17.1  4.9  2.4  7.3  2.4  2.4  39 | |
| Toxicities (multiple answers possible) | Fatigue  Alopecia/erythema  Headaches  Nausea+/-vomiting  Dysphagia/Xerostomia  Gait ataxia  No  NA | 13  9  4  2  3  1  13  11 | 31.7  22  9.8  4.9  7.3  2.4  31.7  26.8 | |
| **Clinical factors** |  | **N** | **%** | |
| Recurrence or metastasis after radiation of primary tumor (n=13) |  | 8 | 61.5 | |
| **Specifications of Radiotherapy** | **Available for N (%)** | **Median** | **Range** | |
| CTV in ccm | 18 (43.9) | 56.85 | 1.6-251.1 | |
| PTV in ccm | 26 (63.4) | 164.4 | 2.9-1565 | |
| Dose in Gray for all tumors | 39 (95.1) | 50 | 16-65.1 | |
| Fractions for all tumors | 30 (73.2) | 25 | 7-33 | |
| Dose in Gray for primary tumors | 13 (100) | 57.6 | 38.5-60 | |
| Fractions for primary tumors | 10 (76.9) | 29 | 7-30 | |
| Dose in Gray for recurrences/metastases | 26 (92.8) | 45 | 16-65.1 | |
| Fractions for recurrences/metastases | 20 (71.4) | 22 | 10-33 | |

NA = Not available, IMRT = Intensity modulated radiotherapy, IGRT = Image guided radiation therapy, VMAT = Volume modulated arc radiotherapy, CTV = Clinical target volume, PTV = planning target volume
